# Toxic effects of trace phenol/guanidine isothiocyanate (P/GI) on cells cultured nearby in covered 96-well plates

**DOI:** 10.1186/s12896-022-00766-2

**Published:** 2022-11-25

**Authors:** Madeline Snedden, Lavisha Singh, Chandrashekara Kyathanahalli, Emmet Hirsch

**Affiliations:** 1 Department of Obstetrics and Gynecology, NorthShore University HealthSystem, 2650 Ridge Ave, Suite 1538, Evanston, IL 60201 USA; 2 Department of Statistics, NorthShore University HealthSystem, Evanston, IL USA; 3 Department of Obstetrics and Gynecology, Pritzker School of Medicine, University of Chicago, Chicago, IL USA

**Keywords:** Phenol and guanidine isothiocyanate, TRIzol, RAW264.7 cells, Time course experiments, Tissue culture

## Abstract

**Background:**

A mixture of phenol and guanidine isothiocyanate (“P/GI”, the principal components of TRIzol™ and similar products) is routinely used to isolate RNA, DNA, and proteins from a single specimen. In time-course experiments of cells grown in tissue culture, replicate wells are often harvested sequentially and compared, with the assumption that in-well lysis and complete aspiration of P/GI has no effect on continuing cultures in nearby wells.

**Methods:**

To test this assumption, we investigated morphology and function of RAW 264.7 cells (an immortalized mouse macrophage cell line) cultured in covered 96-well plates for 4, 8, or 24 h at varying distances from a single control well or a well into which P/GI had been deposited and immediately aspirated completely.

**Results:**

Time- and distance-dependent disruptions resulting from proximity to a single well containing trace residual P/GI were seen in cell morphology (blebbing, cytoplasmic disruption, and accumulation of intracellular vesicles), cell function (pH of culture medium), and expression of genes related to inflammation (*Tnfα*) and autophagy (*Lc3b*). There was no transcriptional change in the anti-apoptotic gene *Mcl1*, nor the pro-apoptotic gene *Hrk*, nor in P/GI-unexposed control cultures. LPS-stimulated cells incubated near P/GI had lower expression of the cytokine *Il6*. These effects were seen as early as 4 h of exposure and at a distance of up to 3 well units from the P/GI-exposed well.

**Conclusions:**

Exposure to trace residual quantities of P/GI in covered tissue culture plates leads to substantial disruption of cell morphology and function in as little as 4 h, possibly through induction of autophagy but not apoptosis. This phenomenon should be considered when planning time-course experiments in multi-well covered tissue culture plates.

**Supplementary Information:**

The online version contains supplementary material available at 10.1186/s12896-022-00766-2.

## Background

96-well culture plates are often used to study cellular responses to various stimuli in vitro. To efficiently recover RNA, DNA, and protein from such specimens, a monophasic solution of phenol and guanidine isothiocyanate (“P/GI”, commercially available as TRIzol™, TRI Reagent™, and others) is often used [1]. In controlled time-course experiments in multi-well tissue culture plates, replicate cultures can be treated with a stimulus at one time and harvested at various times afterwards using P/GI. Phenolic compounds [2] and guanidine compounds [3] are cytotoxic, but the incidental effects of trace amounts of these volatile substances, either separately or in combination, on adjacent cell culture wells (as opposed to direct exposure in culture medium) has not been investigated.

As a chaotrope, P/GI quickly breaks down cellular structures and dissolves cell components when added directly onto the culture dish. It may also induce programmed cell death (apoptosis [3, 4] or autosis [5]) or non-programmed cell death (necrosis [4]). Apoptosis, characterized by cell shrinkage, membrane blebbing, DNA condensation, and fragmentation, is triggered by activation of death receptor-induced extrinsic or cell injury-induced intrinsic pathways [6]. Apoptotic pathway activation may be monitored by evaluating the expression of tumor necrosis factor α (*Tnf*α, a death receptor [7]), induced myeloid cell leukemia cell differentiation protein (*Mcl1*, a pro-survival protein at the intersection of the intrinsic and extrinsic apoptotic pathways [8]), and harakiri (*Hrk*, an intrinsic pro-death protein [8]). Autosis is an autophagy-dependent process initiated upon cellular stress and is characterized by plasma membrane blebbing and loss of cytoplasmic organelles in the absence of chromatin condensation [5]. A progressive accumulation of autophagy-inducing peptides causes the degradation of pro-survival proteins leading to autosis [9]. Changes in the expression of microtubule-associated proteins 1A/1B light chain 3B (*Lc3b*, a key factor in the initiation and maintenance of autophagy), can be an indicator of increased or inhibited autophagic flux [10]. Necrosis is stimulated by cell injury or toxins and leads to cell edema, plasma membrane rupture, loss of cell organelles, and, ultimately, cell death [6]. If no clear pattern of programmed cell death gene expression is detected alongside these morphological changes, necrosis is left as the presumptive cell death mechanism at work.

In the present study, we assessed whether exposure to residual trace amounts of P/GI (i.e. after complete aspiration of P/GI from a single well) affects ongoing cultures in nearby wells in covered 96-well tissue culture plates. We assessed cellular morphology and function, and expression of genes related to inflammation, autophagy, and cell death in RAW 264.7 cells (an immortalized murine macrophage cell line) plated at various distances from a well containing trace residual P/GI for short (4 h)- and long-term (8 h and 24 h) exposures, and in cells stimulated with bacterial lipopolysaccharide (LPS).

## Results

**Fig. 1:**
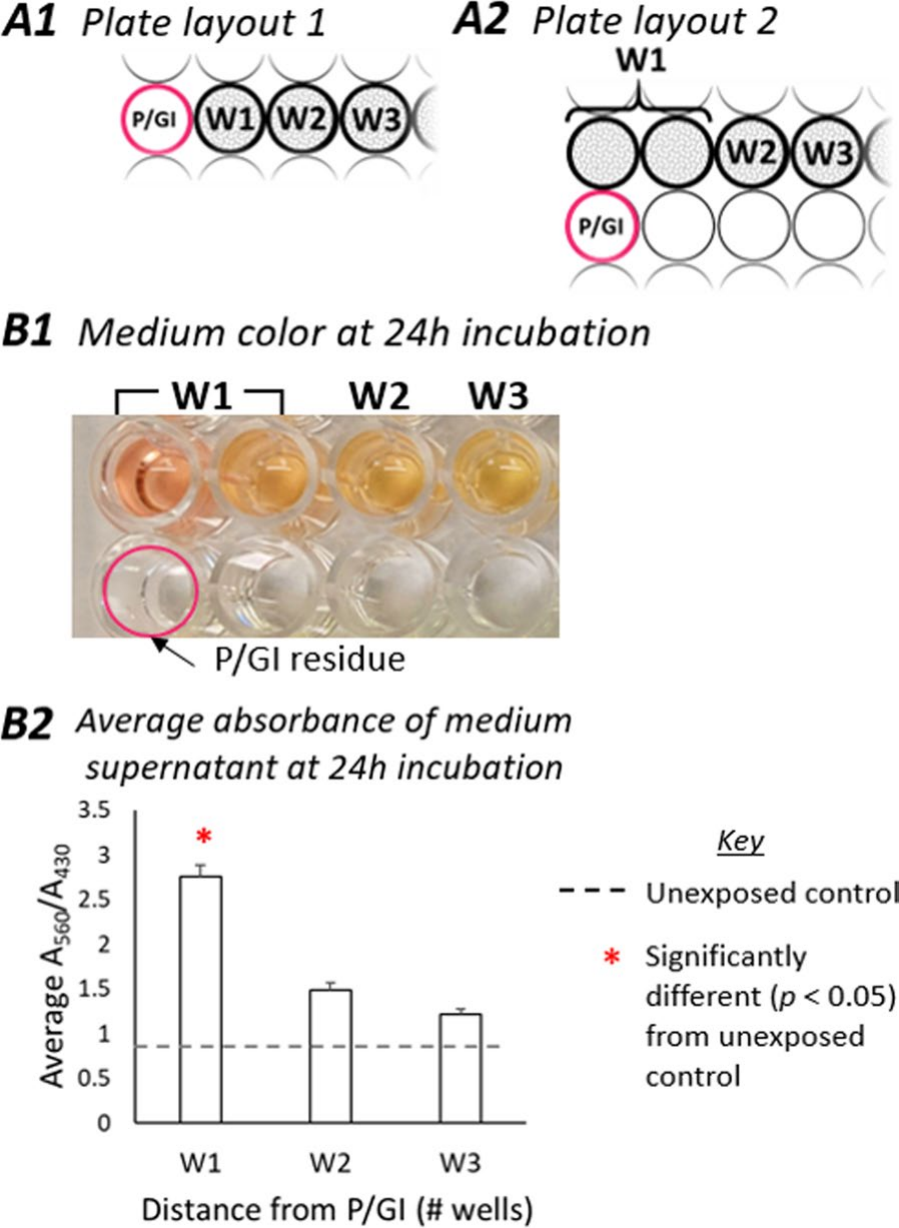
Plate layouts and effect of residual trace P/GI on pH of conditioned medium. Ongoing cell cultures were designated according to their distance (in nearest whole-number well diameter units) from 1 well that briefly contained P/GI. Cells in the well adjacent to trace P/GI residue (i.e. 1 well away) are designated as W1, cells in the next well (2 wells away) as W2, and so on. Cells incubated on a separate 96-well plate without P/GI residue are referred to as “unexposed controls.” The layout depicted in **A1** was used for morphological and gene expression data, and the layout in **A2** was used for culture medium and gene expression data. **B1**) Appearance of culture medium 24 h after exposure to trace residual P/GI (P/GI placed into and immediately aspirated from an empty well). Representative plate from 3 repeated experiments. **B2**) Medium was sampled in duplicate from the cell culture wells and absorbance at 560 nm and 430 nm was measured, where higher ratios between them indicate a redder color and higher pH. For simplicity, values for the two adjacent wells labeled “W1” were averaged. Values show average 560/430 ratio ± standard deviation, which have been normalized so that the average absorbance in unexposed controls equals 1

Results from experiments using two plate layouts (Fig. 1A1, A2), each conducted three times, demonstrate clear differences between cells incubated at various distances (in units of well diameter, termed “W1,” “W2,” W3,” and so on, see Fig. 1 legend) from a well into which P/GI was deposited and then immediately aspirated, and control cells incubated on separate 96-well plates without P/GI residue (“unexposed controls”). Because there were no significant differences between unexposed controls and cells in wells more than 3 wells away from P/GI, we have reported data only from W1-W3.

### Effect of trace P/GI on culture medium pH

Phenol red was added to culture medium as a pH indicator. Phenol red is red–orange at physiologic pH (7.2–7.4), and deviations below 6.8 or above 8.2 appear yellow or fuchsia, respectively [11]. To assess the effect of trace residual P/GI on medium pH, we used photography and spectroscopy to measure the ratio of absorbance at 560 nm (where higher values indicate a redder color, consistent with higher pH) and 430 nm (a reference wavelength). By 24 h after exposure to trace residual P/GI, the medium in W1 had higher 560/430 ratios than unexposed controls (Fig. 1B1, B2), suggesting a less active metabolic state (i.e. diminished production and secretion of acids into conditioned medium). No differences were seen at 8 h (Additional File 1: Fig. S1), or in wells at a greater distance than W1 from the P/GI-treated well.

### Effect of P/GI on cell morphology

**Fig. 2:**
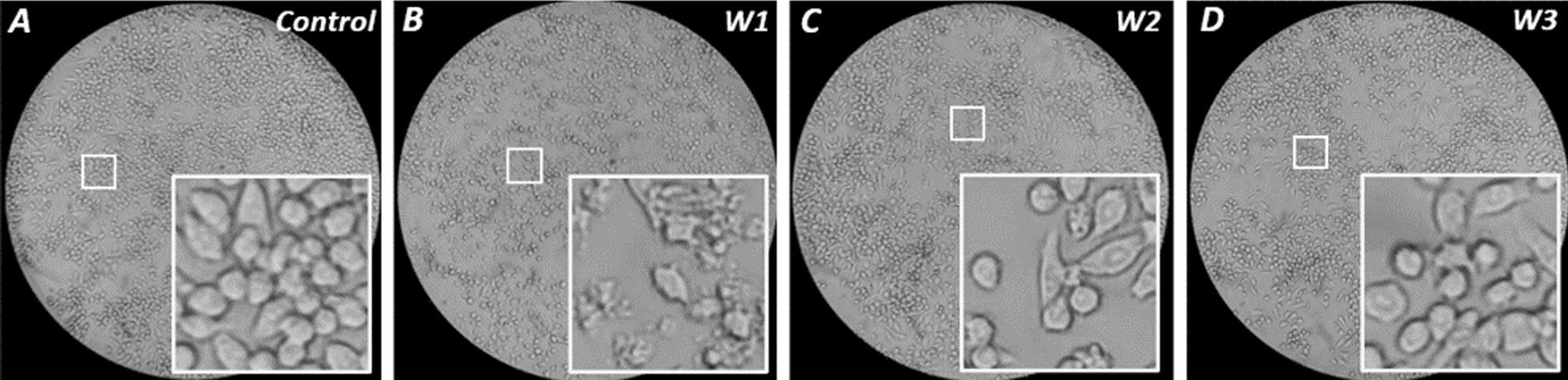
Cell morphology after 24 h P/GI exposure. RAW 264.7 cells at 400X magnification at various distances (W1, W2, or W3) from trace residual P/GI for 24 h (**B**, **C**, and **D**) or time-matched unexposed control (**A**)

We examined whether exposure to trace residual P/GI affects cell morphology by light microscopy. Cells incubated at any distance from P/GI for 4 h were morphologically indistinguishable from time-matched unexposed controls, but by 8 h cells in W1 had various membrane abnormalities, including blebbing and multiple intracellular vesicles (see Additional File 1: Fig. S2). After 24 h, unexposed controls (Fig. 2A) had become near-confluent and displayed diverse morphologies typical of undifferentiated monocytes and M2-polarized macrophages [12]. In contrast, many cells in W1 (Fig. 2B) had a strikingly shrunken appearance and extensive plasma membrane perturbations. Cells in W2 (Fig. 2C) possessed more intracellular vesicles than unexposed controls or cells in W3 (Fig. 2D) or W1, in which the cytoplasm was often too contracted to identify intracellular features.

### The effect of P/GI on cellular pathways: programmed cell death, autophagy, and inflammation

**Fig. 3:**
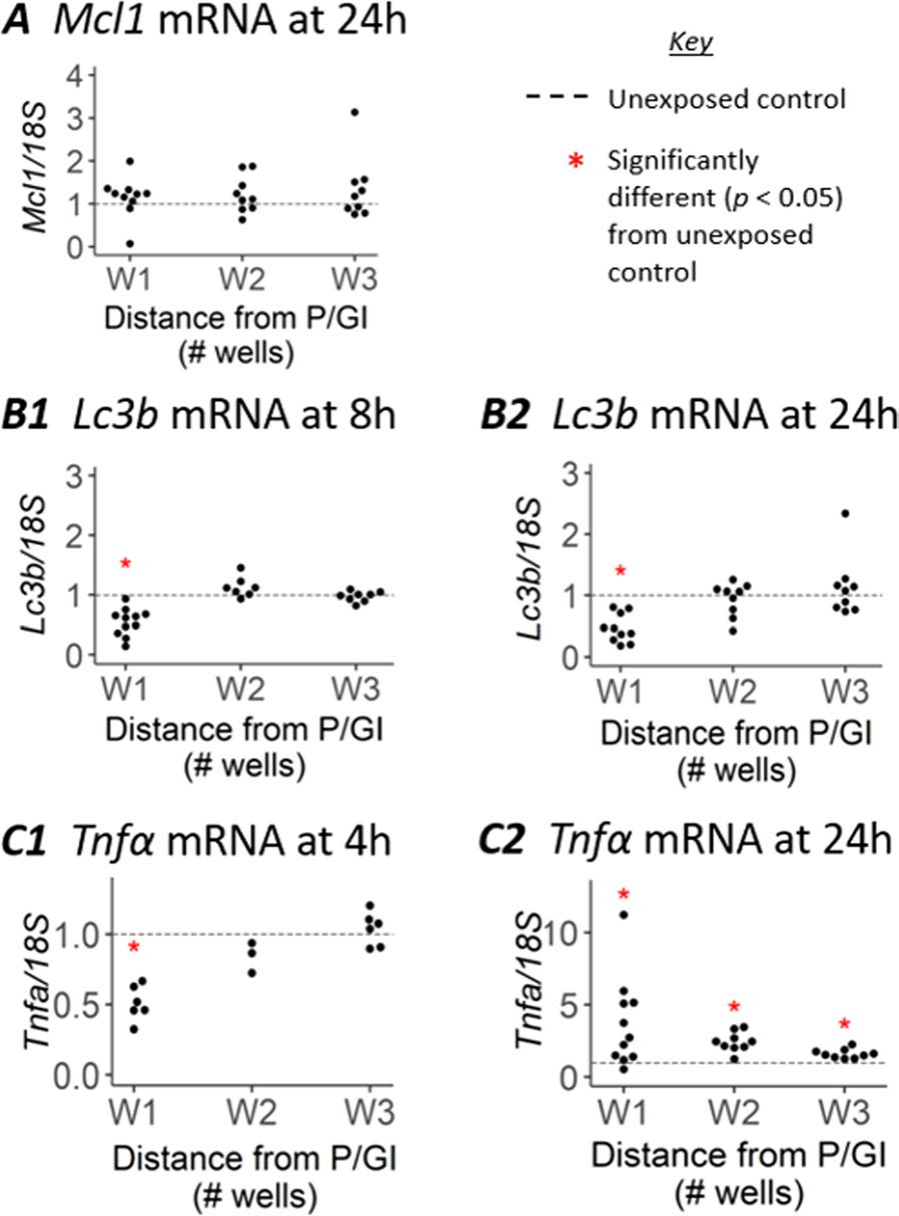
Expression of genes associated with cell survival, autophagy, and inflammation. RT-PCR data measuring expression of mRNA for Mcl1 (**A**), Lc3b (**B**), and Tnfα (**C**) in RAW 264.7 cells cultured at various distances from P/GI residue at the times indicated. Transcripts are normalized so that the average expression in unexposed controls equals 1

The above results (diminished acid production and abnormal morphology) suggest that exposure to trace residual P/GI disrupts normal cell function. To explore whether this disruption might be mediated by changes in apoptotic pathways, the expression of 2 genes was interrogated. *Mcl1* is an anti-apoptotic gene capable of regulating both intrinsic and extrinsic apoptotic pathways [8]. *Hrk* is a pro-apoptotic member of the BH3-only family; when expressed it antagonizes MCL1, thereby allowing apoptosis to occur [8]. Expression of both MCL1 [13] and HRK [14] are under transcriptional control. Real-time polymerase chain reaction (RT-PCR) results for *Mcl1* showed uniform expression at all distances from P/GI, equivalent to *Mcl1* expression in unexposed controls, at all time points tested (24 h data shown in Fig. 3A; see Additional File 1: Fig. S3 for 4 h and 8 h data). *Hrk* was not detected in experimental samples regardless of duration of and distance from P/GI exposure.

We next queried the process of autophagy as a mediator of P/GI’s effects on morphology and acidity of culture medium. Experiments designed specifically to monitor autophagic flux generally capture protein abundance, modifications, and localization [10], but for our purposes—to determine whether autophagy might be impacted in cells near P/GI—mRNA data is sufficient. Transcription of *Lc3b*, an important protein in autophagosome membrane elongation, can be affected hours after cells are exposed to stimuli that produce changes in autophagy [10]. No changes in *Lc3b* were detected at the 4 h time point (see Additional File 1: Fig. S3), but at both 8 h (Fig. 3B1) and 24 h (Fig. 3B2) there are lower levels of *Lc3b* in W1 compared to unexposed controls.

We also assayed expression of *Tnfα*, a gene associated with various processes, including programmed cell death and inflammation [7]. *Tnfα* expression was significantly decreased in W1 at 4 h (Fig. 3C1), but by 24 h (Fig. 3C2) cells from W1—W3 had significantly elevated *Tnfα* transcripts.

### Cells exposed to P/GI have disrupted responses to LPS stimulation

**Fig. 4:**
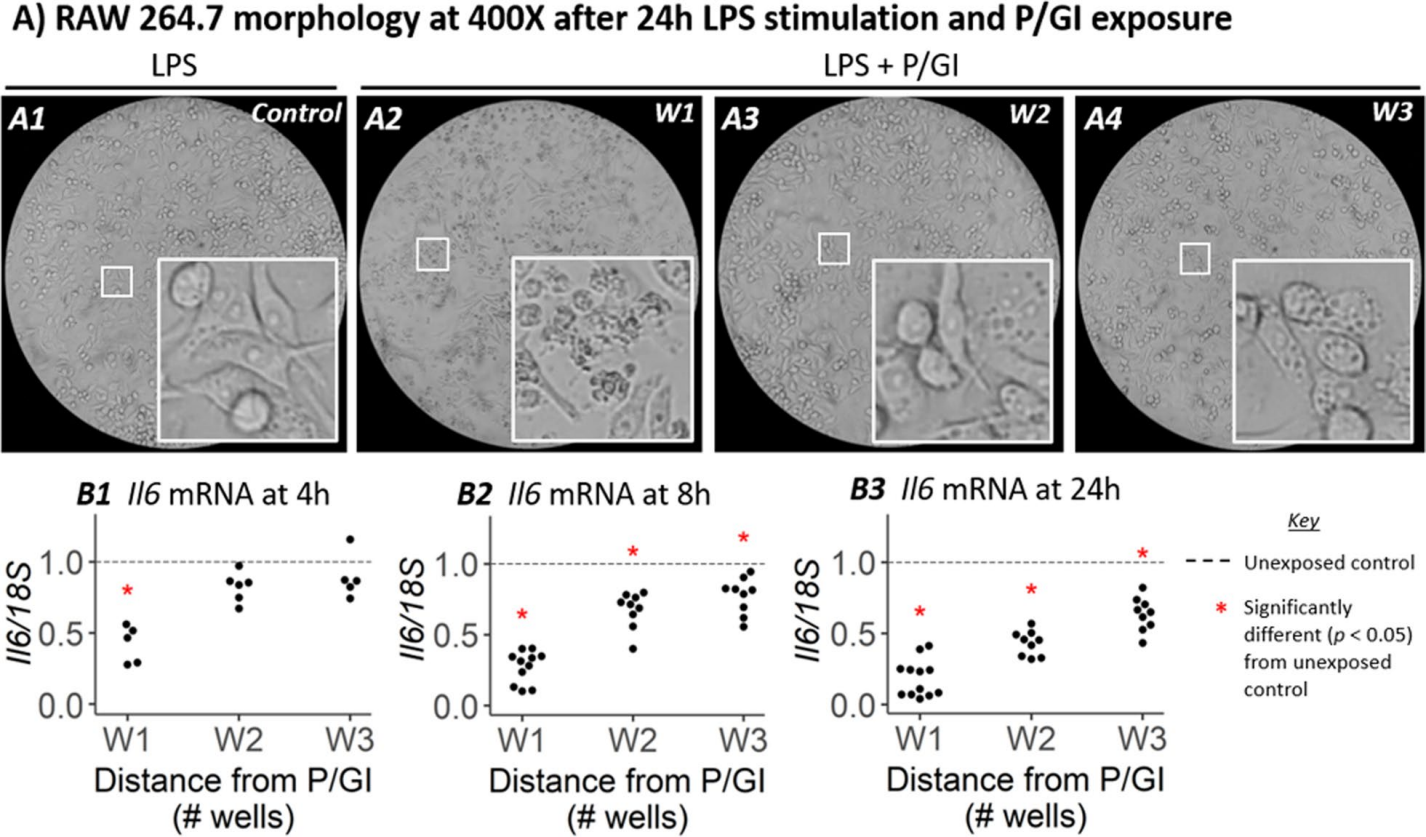
Cell morphology and gene expression in LPS-stimulated cells. **A**) LPS-stimulated RAW 264.7 cells at 400X magnification 24 h after addition and aspiration of P/GI to a single well various distances away (**A2**, **A3**, and **A4**) or time-matched LPS-treated but P/GI-unexposed controls (**A1**). **B**) RT-PCR data measuring expression of Il6 transcripts in RAW 264.7 cells cultured at various distances from P/GI residue at 4 h, 8 h, and 24 h after LPS exposure (**B1**, **B2**, and **B3**, respectively). Transcripts are normalized so that the average expression in P/GI-unexposed controls equals 1

By running parallel experiments in cells stimulated with LPS, we were able to evaluate functional changes resulting from a proinflammatory stimulus in cells at various distances from P/GI. Compared to cells not treated with LPS, cells treated with LPS for 24 h but unexposed to P/GI (Fig. 4A1) were larger, flatter, and had well-defined intracellular vesicles—hallmarks of classic M1 polarization [12]. LPS-stimulated cells cultured near P/GI residue displayed aberrant morphology (including shrunken cytoplasm, blebbing, and intracellular vesicles) at 4 h and 8 h (Additional File 1: Fig. S4), and by 24 h the cells in W1 (Fig. 4A2) had contracted cytoplasm and abundant blebbing. This phenotype was observed occasionally in W2 (Fig. 4A3) and W3 (Fig. 4A4); however, frequently these changes were obscured by the abundance of intracellular vesicles.

To assess the impact of P/GI on responses to an inflammatory stimulus, we measured interleukin 6 mRNA (*Il6*), a commonly used marker of the inflammatory response to LPS [12]. *Il6* is transcribed at high levels in LPS-stimulated macrophages, which we confirmed in P/GI-unexposed controls. P/GI produced a time- and distance-dependent diminishment of *Il6* expression with LPS stimulation. After 4 h stimulation with LPS on culture plates containing residual trace P/GI, cells in W1 had lower *Il6* (Fig. 4B1). At 8 h and 24 h suppression of *Il6* transcription was more pronounced in W1 and became apparent in W2 and W3 as well (Fig. 4B2, B3). Expression patterns of other genes (*Tnfα*, *Lc3b,* and *Mcl1*) was not altered by LPS (see Additional File 1: Fig. S5).

## Discussion

### Implications

Culture medium spectroscopy results (i.e. higher pH) suggest a less active metabolic state induced by proximity to trace residual P/GI, as metabolically active cells secrete acids continually into the medium [11]. This is supported by diminished expression of *Lc3b* (indicating departure from physiologic autophagy [10]) and *Tnfα* (which plays a role in programmed cell death, bioenergetics, and initiating and sustaining inflammation in macrophages responding to damaged cells or pathogens [7, 15]). The delayed increase observed in *Tnfα* expression 24 h after exposure to P/GI may reflect a compensatory response to the initial insult. These disruptions in cell function are correlated with diminished responsiveness to LPS stimulation and induction of marked abnormalities in cell morphology [12].

A lack of modulation of *Mcl1* and *Hrk* suggests that their associated programmed cell death pathways (apoptosis and autosis among them [9, 16]) may not be responsible for these apparent changes. Nevertheless, lack of transcriptional changes does not rule out the possibility that post-translational mechanisms mediate these processes.

Overall, these results suggest that even minute quantities of P/GI meaningfully disrupt normal cell function in a process potentially mediated by altered autophagy.

### Limitations

While the data demonstrate that trace P/GI is capable of affecting nearby macrophage morphology and function, the mechanisms behind these effects are unclear. This might be addressed by increasing the number of genes interrogated, adding protein data, and exploring P/GI’s effects on a second cell line, which were not done in this limited study. Additionally, we are unable to extrapolate how far-ranging these effects may be when multiple wells per plate contain trace residual P/GI (as expected in an experiment with multiple replicates per plate), because it would be difficult to characterize a well’s distance from the points of P/GI application.

## Conclusions

Trace amounts of P/GI remaining in wells from which it has been immediately and fully aspirated are capable of disrupting cell morphology and function (including autophagy and inflammation) in macrophages cultured nearby on the same covered multi-well plate in a time- and distance-dependent fashion. Changes become apparent as early as 4 h after exposure, but it is likely that the molecular processes underlying these phenomena begin even earlier. In our experiments, no changes were observed in ongoing cultures distanced more than 3 wells away from a single well containing P/GI residue. However, it is possible that such changes occur in select processes even at large distances. Given the volatile nature of P/GI [2], fumes from trace P/GI left over after lysing cells at 1 collection point may affect other cells on the same culture plate at a later collection point. To avoid the confounding effects of even minute quantities of P/GI, laboratories should take these observations into account when designing time-course experiments in tissue culture plates.

## Methods

### Cell culture

RAW 264.7 cells (ATCC, Manassas, VA) were cultured in Dulbecco’s modified eagle medium (Gibco, Waltham, MA) + 10% fetal bovine serum (Gibco) in all experiments. Cells were seeded 10^5^ per well in 200 μL medium in 96-well plates, covered, and cultured overnight at 37 °C in a humidified atmosphere with 5% CO_2_. After overnight culture, the medium was replaced, and for each of three time points (4, 8, or 24 h), plates were exposed to either residual P/GI or control (an empty well). The “exposure” entailed filling a single well with 100 μL TRI Reagent™ (Molecular Research Center, Cincinnati, OH), pipetting up and down, and immediately completely aspirating the solution. In some experiments, cells were treated concurrently with 0.2 ng/mL LPS to assess P/GI’s effect on the induction of inflammatory genes.

Cell morphology (using plate layout 1, Fig. 1A1) was observed under a light microscope (Zeiss, Jena, Germany) at 400X magnification at the end of each incubation period.

### Cell culture medium color changes

Changes in the pH of the cell culture medium (plate layout 2, Fig. 1A2) were indirectly measured using color changes of phenol red (an acid–base indicator) in the culture medium. Briefly, 50 µL of the cell culture medium was sampled in duplicate from each well 30 min before the end of the incubation period, and absorbance was measured at 560 nm (with greater absorbance indicative of a higher pH) and 430 nm (a reference wavelength) using a SpectraMax M2e (Molecular Devices, San Jose, CA) [11]. Results from both W1 wells in each experiment (Fig. 1B1) were averaged for data analysis (Fig. 1B2).

### Gene expression analysis

After incubation, medium was aspirated, and cells were directly lysed in TRI Reagent™. RNA was extracted according to the manufacturer’s instructions and quantified using a NanoDrop 2000 (ThermoFisher, Waltham, MA). cDNA was synthesized using qScript cDNA SuperMix (Quantabio, Beverly, MA), with 100 ng RNA per reaction. RT-PCR was performed using TaqMan Gene Expression MasterMix (ThermoFisher) on the StepOne Plus (Applied Biosystems, Waltham, MA) with the following pre-validated primer/probe assay mixes: 18 s rRNA (Hs03003631_m1), *Il6 (*Mm00446190_m1), *Tnfα* (Mm00443258_m1), *Lc3b* (Mm00782868_sH), *Mcl1* (Mm01257531_g1), and *Hrk* (Mm01208086_m1), all from ThermoFisher. Gene expression was quantified using the ddCt method for RT-PCR, in which genes of interest are normalized against a housekeeping gene (18S).

### Statistical analysis

The culture medium 560/430 ratios and log-transformed gene expression measurements (*Il6*, *Lc3b*, *Mcl1*, *Tnfα*, normalized against *18S*) of cells cultured at various distances (W1, W2, W3) from PG/I for 4 h, 8 h, or 24 h were compared to time-matched unexposed controls using the non-parametric Dwass-Steel-Critchlow-Fligner (DSCF) test for multiple pairwise comparisons. P-values less than 0.05 were considered statistically significant. All statistical analyses were performed using SAS version 9.4 (SAS Institute Inc., Cary, NC, USA) or Excel 2016 (Microsoft, Redmond, WA, USA).

## Supplementary Information

**Additional file 1.** Supplemental images and graphs supporting secondary findings.

## Data Availability

All data generated or analyzed during this study are included in this published article and its supplementary information files.

## Abbreviations

Hrk: Harakiri or DP5
Il: Interleukin
Lc3b: Microtubule-associated proteins 1A/1B light chain 3B, aka MAP1LC3B or ATG8
LPS: Lipopolysaccharide
Mcl1: Induced myeloid cell leukemia cell differentiation protein or BCL2L3
P/GI: Phenol and guanidine isothiocyanate
Tnfα: Tumor necrosis factor α
