# Supplementary material for: Toxic effects of trace phenol/guanidine isothiocyanate (P/GI) on cells cultured nearby in covered 96-well plates
Source: BMC Biotechnol. 2022 Nov 25;22:35. doi: 10.1186/s12896-022-00766-2 (PMC9700959; doi:10.1186/s12896-022-00766-2)
Supplement: Supplementary file 1 — Additional file 1. Supplemental images and graphs supporting secondary findings. [file 12896_2022_766_MOESM1_ESM.pptx]

## Slide 1
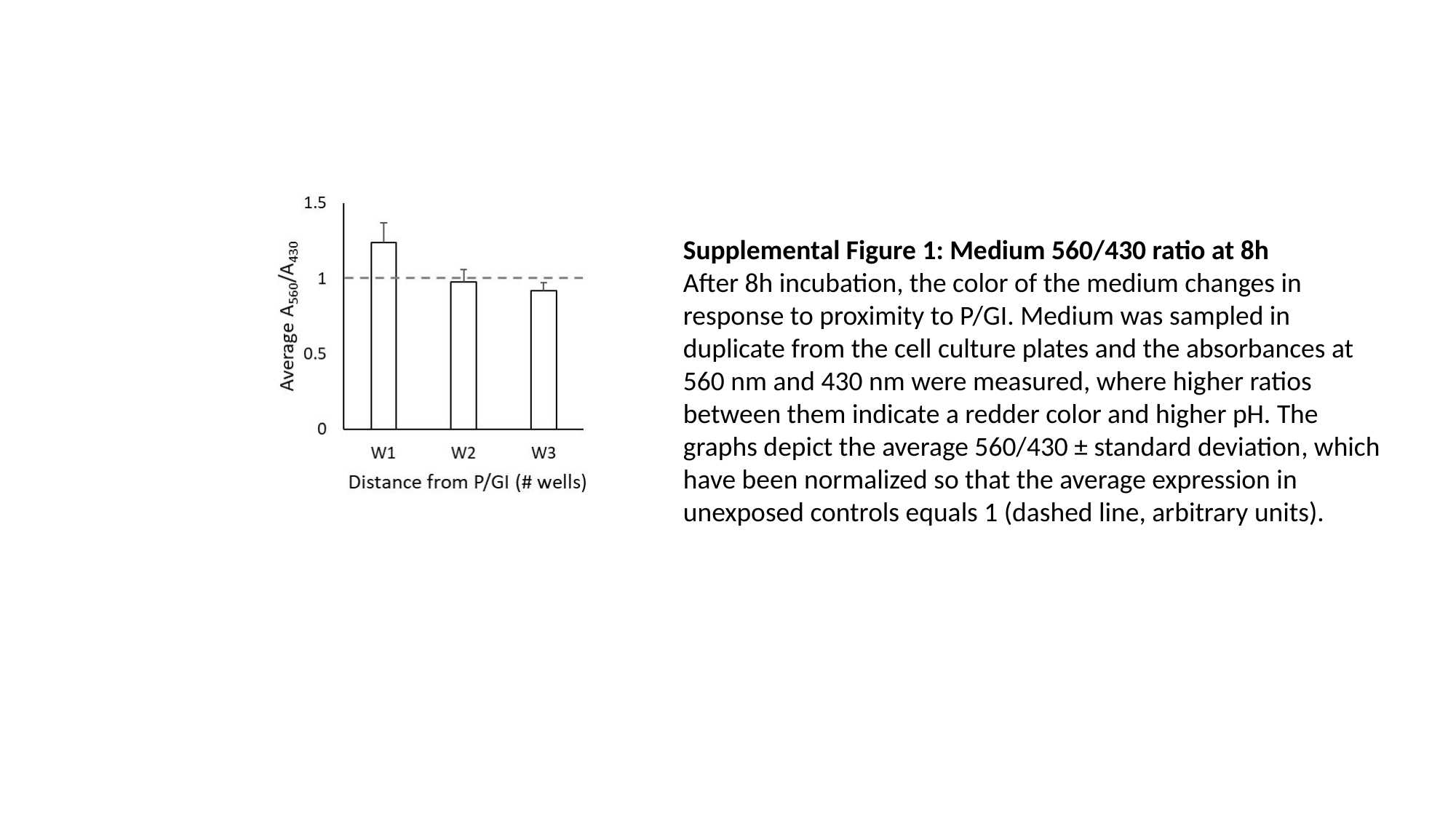

Supplemental Figure 1: Medium 560/430 ratio at 8h
After 8h incubation, the color of the medium changes in response to proximity to P/GI. Medium was sampled in duplicate from the cell culture plates and the absorbances at 560 nm and 430 nm were measured, where higher ratios between them indicate a redder color and higher pH. The graphs depict the average 560/430 ± standard deviation, which have been normalized so that the average expression in unexposed controls equals 1 (dashed line, arbitrary units).

## Slide 2
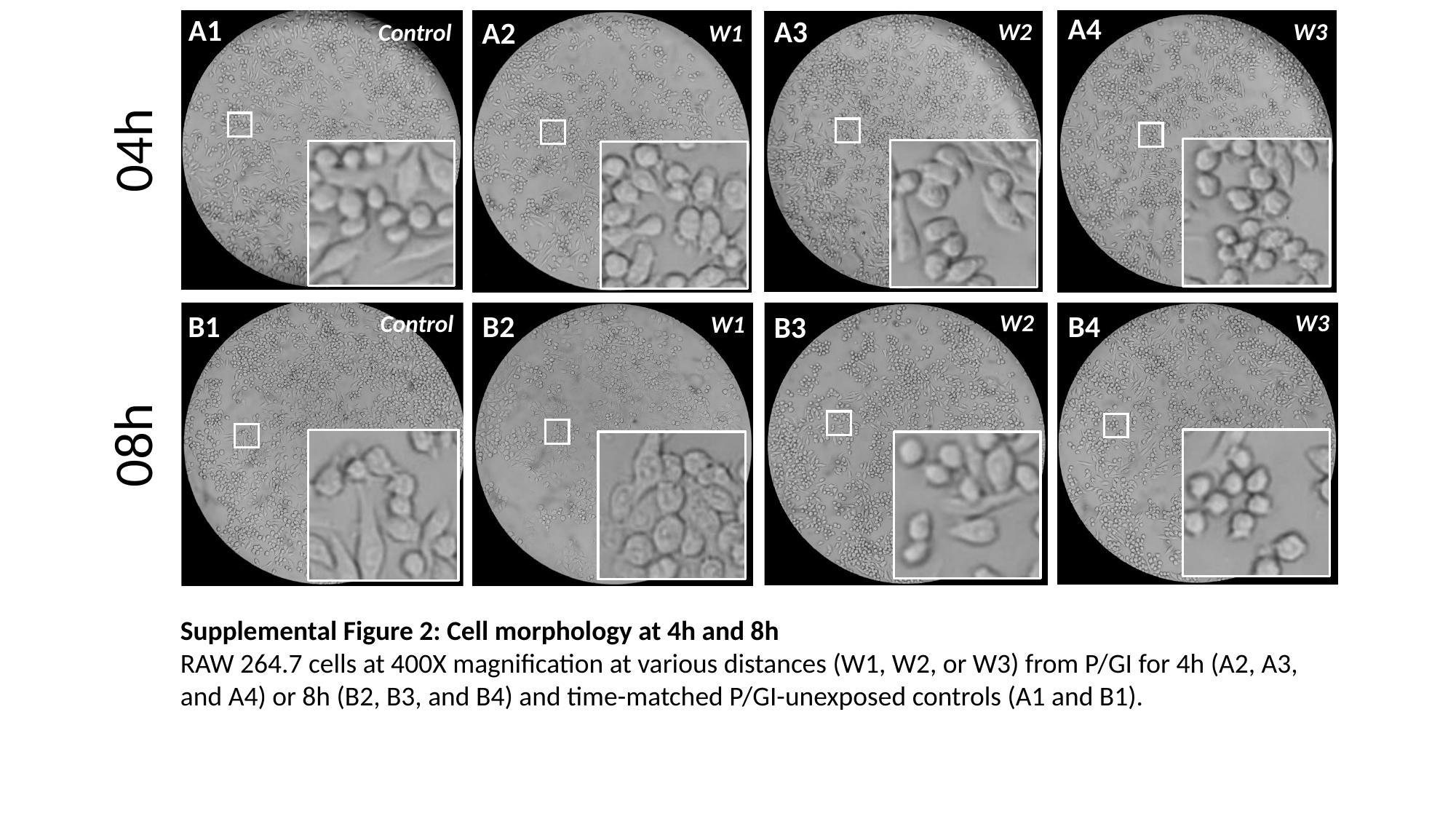

A4
A1
A3
A2
W2
W3
Control
W1
04h
B1
B4
B2
W2
W3
B3
Control
W1
08h
Supplemental Figure 2: Cell morphology at 4h and 8h
RAW 264.7 cells at 400X magnification at various distances (W1, W2, or W3) from P/GI for 4h (A2, A3, and A4) or 8h (B2, B3, and B4) and time-matched P/GI-unexposed controls (A1 and B1).

## Slide 3
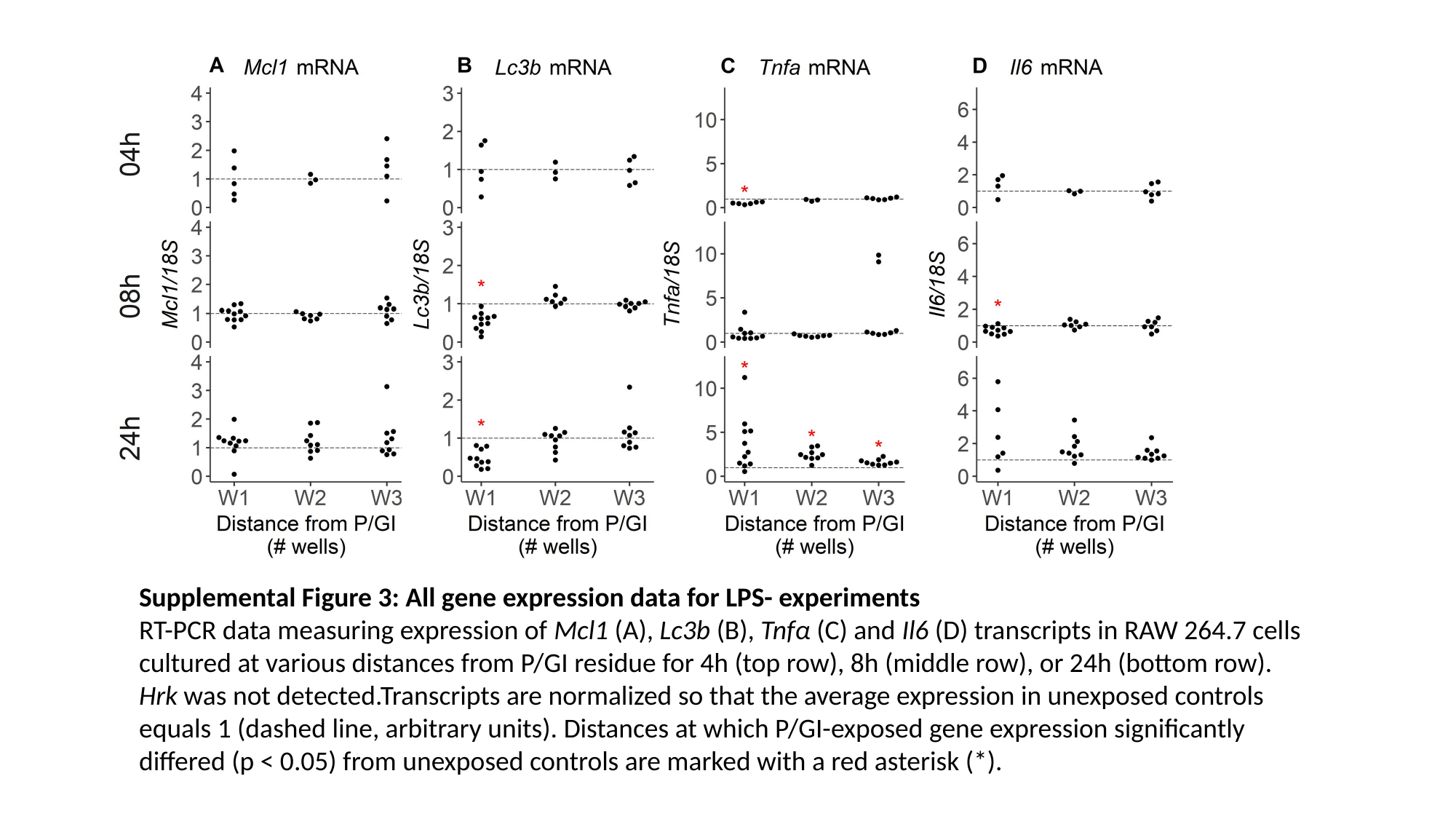

Supplemental Figure 3: All gene expression data for LPS- experiments
RT-PCR data measuring expression of Mcl1 (A), Lc3b (B), Tnfα (C) and Il6 (D) transcripts in RAW 264.7 cells cultured at various distances from P/GI residue for 4h (top row), 8h (middle row), or 24h (bottom row). Hrk was not detected.Transcripts are normalized so that the average expression in unexposed controls equals 1 (dashed line, arbitrary units). Distances at which P/GI-exposed gene expression significantly differed (p < 0.05) from unexposed controls are marked with a red asterisk (*).

## Slide 4
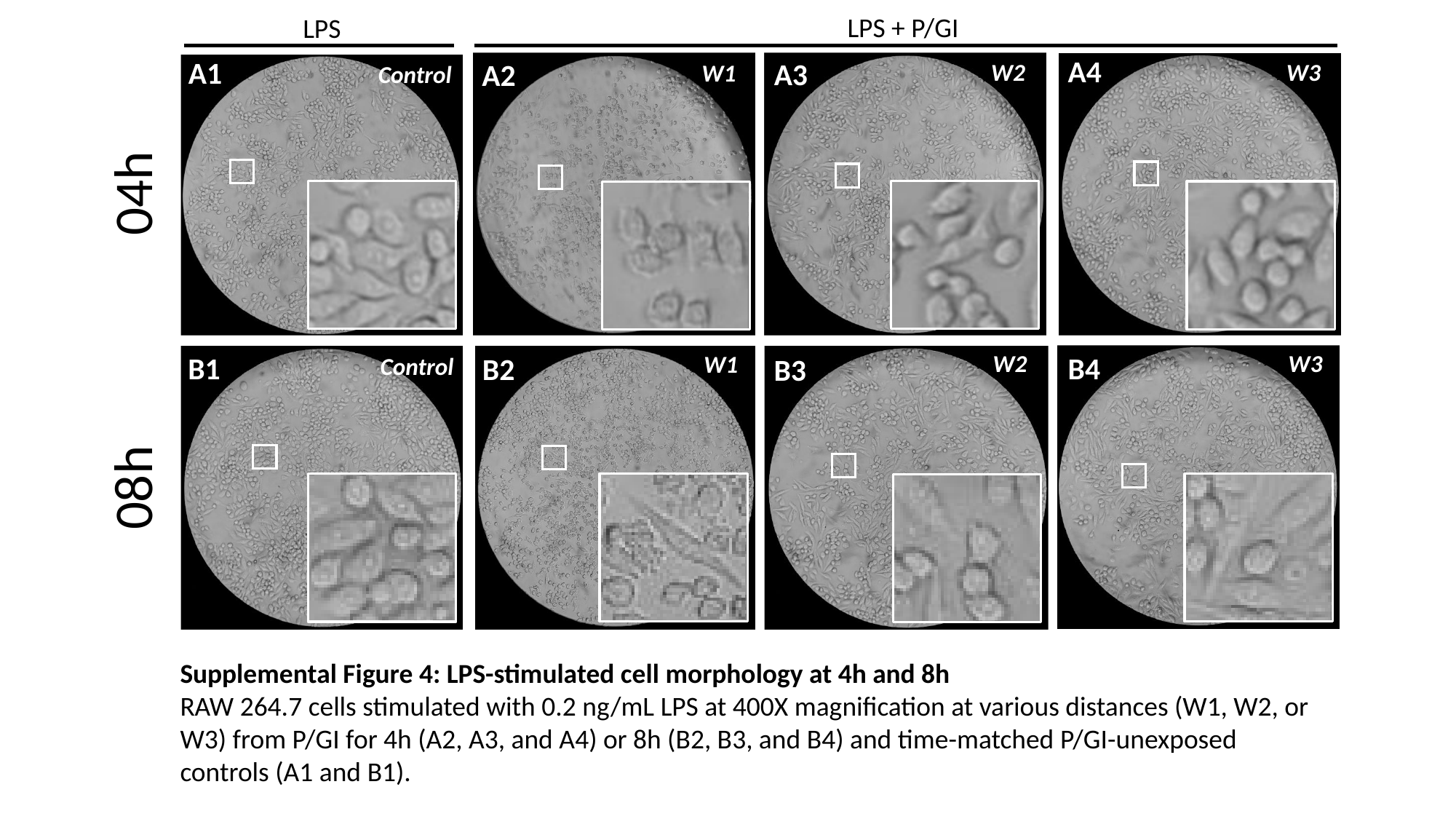

LPS + P/GI
LPS
A4
A1
A3
A2
W2
W3
W1
Control
04h
W2
W3
W1
B1
B4
B2
B3
Control
08h
Supplemental Figure 4: LPS-stimulated cell morphology at 4h and 8h
RAW 264.7 cells stimulated with 0.2 ng/mL LPS at 400X magnification at various distances (W1, W2, or W3) from P/GI for 4h (A2, A3, and A4) or 8h (B2, B3, and B4) and time-matched P/GI-unexposed controls (A1 and B1).

## Slide 5
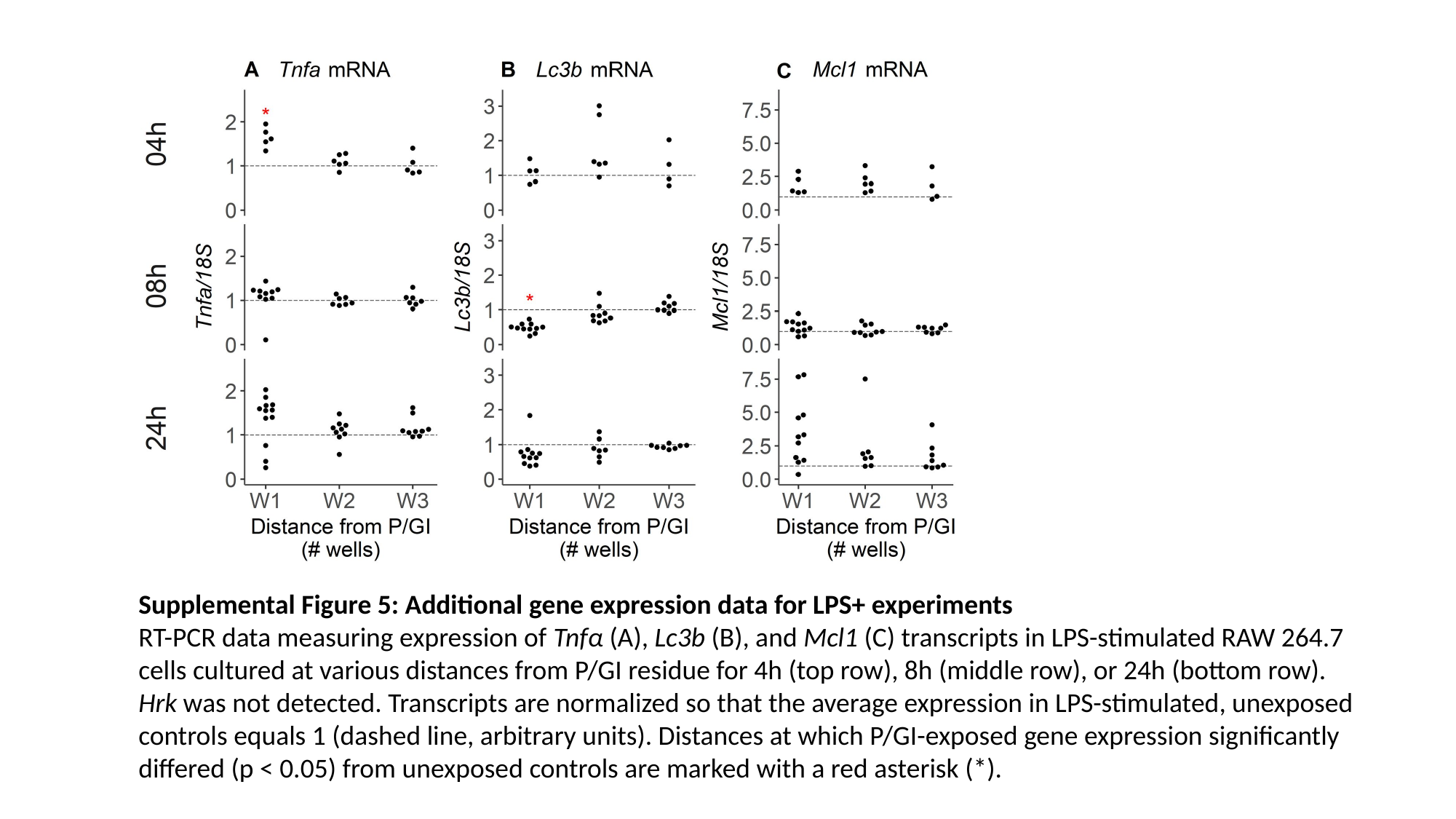

Supplemental Figure 5: Additional gene expression data for LPS+ experiments
RT-PCR data measuring expression of Tnfα (A), Lc3b (B), and Mcl1 (C) transcripts in LPS-stimulated RAW 264.7 cells cultured at various distances from P/GI residue for 4h (top row), 8h (middle row), or 24h (bottom row). Hrk was not detected. Transcripts are normalized so that the average expression in LPS-stimulated, unexposed controls equals 1 (dashed line, arbitrary units). Distances at which P/GI-exposed gene expression significantly differed (p < 0.05) from unexposed controls are marked with a red asterisk (*).
